# Unusual Regulation of a Leaderless Operon Involved in the Catabolism of Dimethylsulfoniopropionate in *Rhodobacter sphaeroides*

**DOI:** 10.1371/journal.pone.0015972

**Published:** 2011-01-07

**Authors:** Matthew J. Sullivan, Andrew R. J. Curson, Neil Shearer, Jonathan D. Todd, Robert T. Green, Andrew W. B. Johnston

**Affiliations:** 1 School of Biological Sciences, University of East Anglia, Norwich Research Park, Norwich, United Kingdom; 2 Institute of Food Research, Norwich Research Park, Norwich, United Kingdom; Auburn University, United States of America

## Abstract

*Rhodobacter sphaeroides* strain 2.4.1 is a widely studied bacterium that has recently been shown to cleave the abundant marine anti-stress molecule dimethylsulfoniopropionate (DMSP) into acrylate plus gaseous dimethyl sulfide. It does so by using a lyase encoded by *dddL,* the promoter-distal gene of a three-gene operon, *acuR-acuI-dddL*. Transcription of the operon was enhanced when cells were pre-grown with the substrate DMSP, but this induction is indirect, and requires the conversion of DMSP to the product acrylate, the *bona fide* co-inducer. This regulation is mediated by the product of the promoter-proximal gene *acuR,* a transcriptional regulator in the TetR family. AcuR represses the operon in the absence of acrylate, but this is relieved by the presence of the co-inducer. Another unusual regulatory feature is that the *acuR-acuI-dddL* mRNA transcript is leaderless, such that *acuR* lacks a Shine-Dalgarno ribosomal binding site and 5′-UTR, and is translated at a lower level compared to the downstream genes. This regulatory unit may be quite widespread in bacteria, since several other taxonomically diverse lineages have adjacent *acuR-*like and *acuI-*like genes; these operons also have no 5′ leader sequences or ribosomal binding sites and their predicted *cis-*acting regulatory sequences resemble those of *R. sphaeroides acuR-acuI-dddL.*

## Introduction

Dimethylsulfoniopropionate (DMSP) is an abundant (∼10^9^ tons produced worldwide, each year) compatible solute that is made by many diverse marine phytoplankton, some macroalgal seaweeds and a few angiosperms [1]. Its exact function is still not clear; it may act as an osmoprotectant and/or anti-stress molecule in response to oxidative damage or UV radiation (see [2]). Some DMSP-producing algae themselves can catabolise DMSP [3], [4] and on senescence or viral lysis, the DMSP that is released from these algae can be catabolised by various marine bacteria and by some fungi [5], [6].

Some of these catabolic pathways generate dimethyl sulfide (DMS), an important volatile in its own right. Approximately 30 million tons (∼10% of the total) of this DMS escapes each year from the surface layers of the sea, representing the principal form by which biogenic sulfur is transferred to the atmosphere from the oceans [7]. Importantly, DMS oxidation products (e.g. sulfate) act as cloud condensation nuclei, with effects on albedo, reflecting radiation back to space [8]. DMS is also a potent chemo-attractant for potential food supplies of marine animals, such as crustaceans (Copepods), seabirds (penguins, shearwaters, petrels) and mammals (harbour seals) [9], [10], [11], [12].

Bacteria can catabolise DMSP in several different ways. Globally, the most important pathway involves demethylation of DMSP to 3-*S*-methylmercaptopropionate (MMPA) and thence to methanethiol and into general metabolism [13], [14]. The *dmdA* gene, which encodes a DMSP demethylase, occurs in several marine α-Proteobacteria and is widespread in the metagenomes of marine bacteria [15], [16]. However, this demethylation pathway does not liberate DMS.

Recent genetic studies have revealed several very different ways in which different lineages of bacteria (and some fungi) release DMS during DMSP catabolism, a phenotype termed Ddd^+^ (DMSP-dependent DMS). This may explain the biochemical and physiological heterogeneity seen in earlier studies on this process [5]. Four different gene products, termed DddD, DddL, DddP and DddQ, were found in various marine bacteria [17], [18], [19], [20], [21], [22]. In all four cases, the corresponding cloned genes, *dddD, dddL*, *dddP* and *dddQ*, could each confer a Ddd^+^ phenotype to *Escherichia coli*.

The subject of this study, DddL, is a “DMSP lyase” [21], the generic term used to describe enzymes that cleave DMSP at its γ-carbon atom to release DMS plus acrylate and a proton [5]. Initially identified in *Sulfitobacter* sp. EE-36, there are close homologues of DddL (∼65% identical at the amino acid level) in related α-Proteobacteria in the *Rhodobacteraceae* (*Loktanella*, *Oceanicola*, *Stappia* and *Rhodobacter*), and in *Fulvimarina,* in the closely related *Aurantimonadaceae* family. To date, all the strains tested that had *dddL* in their genomes had a Ddd^+^ phenotype [21].

DddL was previously annotated as a Domain of Unknown Function, and has no sequence similarity to DddD or DddP, which are respectively in the families of Class III CoA-transferases and M24 peptidases, being found in other bacteria and, in the case of *dddP,* in some Ascomycete fungi [17], [18].

The finding of a DddL DMSP lyase in some strains of *Rhodobacter sphaeroides* was initially surprising, because this species has a long genetic and biochemical history, but had not previously been suspected of making DMS. Of the three *R. sphaeroides* strains whose genomes were sequenced, two (strains 2.4.1 and ATCC17029) had a Ddd^+^ phenotype, but the third, strain ATCC17025, did not [21]. Consistent with this, strains 2.4.1 and ATCC17029 have a 60 kb genomic region that includes *dddL,* but this is missing from strain ATCC17025 [23]. All three strains were isolated around 1935 by van Niel [24] at the Hopkins Laboratory, Monterrey, but their environmental origin(s), whether marine or freshwater, are unknown (Howard Nash, personal communication).

Here, we examined the expression of the operon containing *R. sphaeroides dddL*, revealing unusual features about its regulation.

## Results

**Figure 1:**
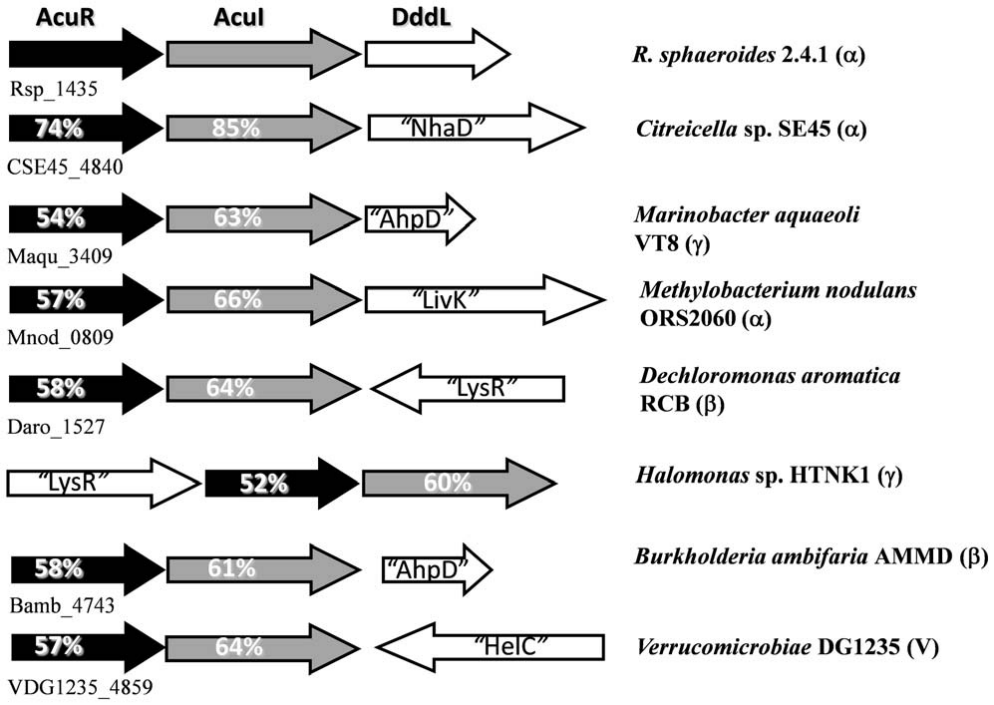
Genomic contexts of *acuR*-like and *acuI-*like genes in *Rhodobacter sphaeroides* and other bacterial species. Examples of adjacent *acuR*-like (black), *acuI*-like (grey) genes are shown for *R. sphaeroides* strain 2.4.1 and for others in the alpha (α), beta (β), and gamma (γ) Proteobacteria and in the Verrucomicrobia phylum (V). Locus tags correspond to the *acuR*-like genes. Percentage identities of the gene products to AcuR and AcuI of *R. sphaeroides* strain 2.4.1 are embedded within the individual genes. Also shown are the polypeptide families encoded by the adjacent genes as follows: AhpD  =  Pfam PF02627; Carboxymuconolactone decarboxylase family. NhaD  =  Pfam PF00939; sodium:sulfate symporter trans-membrane region. LivK  =  Pfam PF01094; extracellular ligand-binding domain of a wide range of receptors. LysR  =  Pfam PF00126; bacterial regulatory helix-turn-helix protein, LysR family. HelC  =  Pfam PF00271; helicase conserved C-terminal domain.

In nearly all bacteria that contain *dddL,* this gene is in a one-gene transcriptional unit, as judged by the positions and orientations of the flanking genes. However, in *R. sphaeroides* strain 2.4.1, *dddL* (locus tag RSP_1433) is the promoter-distal gene of a three-gene operon, as shown below (**Figures 1** **and** **2**). The promoter-proximal gene (RSP_1435), termed *acuR*, is situated 15 bp upstream of the start of RSP_1434, which we term *acuI* (the “*acu*” nomenclature refers to **ac**rylate **u**tilization – see below). All three genes also occur in the same relative location in the genome of the Ddd^+^ *R. sphaeroides* strain ATCC17029, but are absent from that of *R. sphaeroides* ATCC17025, which does not catabolise DMSP [21].

**Figure 2:**
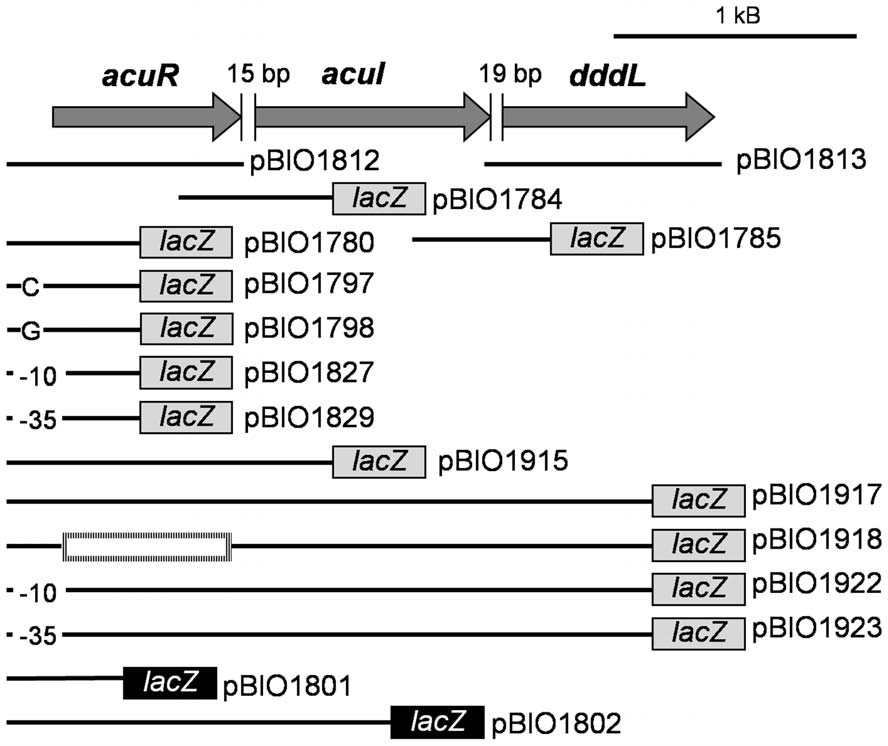
Dimensions of cloned genes and the transcriptional and translational reporter fusions of the *acuR-acuI-dddL* region of *Rhodobacter sphaeroides* 2.4.1. The *acuR, acuI* and *dddL* genes are represented by thick grey arrows, with the sizes of the intergenic gaps shown in base pairs. The dimensions of the cloned *R. sphaeroides* DNA in the various “pBIO” plasmids are represented as black lines. For the *lacZ-*transcriptional fusions in pMP220, the *lacZ* reporters are shown against grey backgrounds. With the translational fusions in pIJ1363, they are shown in black backgrounds. The in-frame deletion in *acuR* in pBIO1918 is shown as a box. Single base pair mutations in the operator in plasmids pBIO1797 and pBIO1798 are indicated, as are those in the −10 (pBIO1827, pBIO1922) and −35 (pBIO1829, pBIO1923) promoter motifs. Dimensions of the fragments that contain intact *acuR* and *dddL* genes (pBIO1812 and pBIO1813 respectively), each cloned in a wide host-range vector, (pOT2 or pKT230) are shown.

AcuR is in the TetR/AcrR family of bacterial transcriptional regulators (COG1309), being 34% identical to *E. coli* AcrR, which down-regulates the multidrug efflux pump AcrAB [25]. AcuI is in the large family of Zn-dependent oxido-reductases (COG0604).

### Induction of DMS production in *Rhodobacter sphaeroides* 2.4.1

DMSP-dependent DMS production had been shown to be weakly increased (∼1.4-fold) when the *dddL-*containing strain *Sulfitobacter* sp. EE36 was pre-grown with 5 mM DMSP [21]. We assayed DMS production in *R. sphaeroides* 2.4.1 and found that this activity was also modestly enhanced (∼3-fold) by DMSP, compared to cells grown in the absence of any inducer (data not shown).

In other Ddd^+^ bacteria, DMS production can be induced not only by the DMSP substrate, but, more unusually, by some products of DMSP catabolism. For example, the Ddd^+^ phenotype of *Halomonas* HTNK1 was enhanced when cells were grown in the presence of 3-hydroxypropionate (3HP), which is a DMSP catabolite in *Halomonas*. This induction was due to enhanced transcription of the *ddd* genes when *Halomonas* was grown with 3HP [19]. To see if a DMSP catabolite was an effective inducer in *R. sphaeroides,* cells of strain 2.4.1 were grown in M9 minimal medium with succinate as C source in the presence of the catabolite acrylate (1 mM) prior to assaying DMSP-dependent DMS production. Strikingly, this caused a large (9-fold) induction of DMS production. In similar assays, 3HP did not act as co-inducer, consistent with the fact that this is not a primary catabolite of the *R. sphaeroides* DMSP-lyase DddL pathway.

We also examined if acrylate induced DMSP-dependent DMS production in some other *dddL-*containing bacteria that have a Ddd^+^ phenotype [21]. In contrast to *R. sphaeroides,* acrylate did not act as a co-inducer of DMS production in *Sulfitobacter* EE-36, *Loktanella vestfoldensis* SKA53, *Stappia aggregata* IAM 12614 and *Fulvimarina pelagi* HTCC2506 (data not shown).

### Induction of *acuR-acuI-dddL* transcription by acrylate and evidence for *trans*-acting regulation

To test if induction of DMSP-dependent DMS production in *R. sphaeroides* 2.4.1 was due to enhanced expression of the *acuR-acuI-dddL* genes, three *lacZ* transcriptional fusion plasmids were made. In these, fragments that included the predicted (and then confirmed – see below) promoter and operator of the *acuR-acuI-dddL* operon were cloned into the low copy-number wide host-range *lacZ* reporter plasmid pMP220 [26]. In these plasmids, pBIO1780, pBIO1915 and pBIO1917, the 3′ ends of the cloned DNA were in *acuR, acuI* and *dddL* respectively (**Figure 2**).

**Figure 3:**
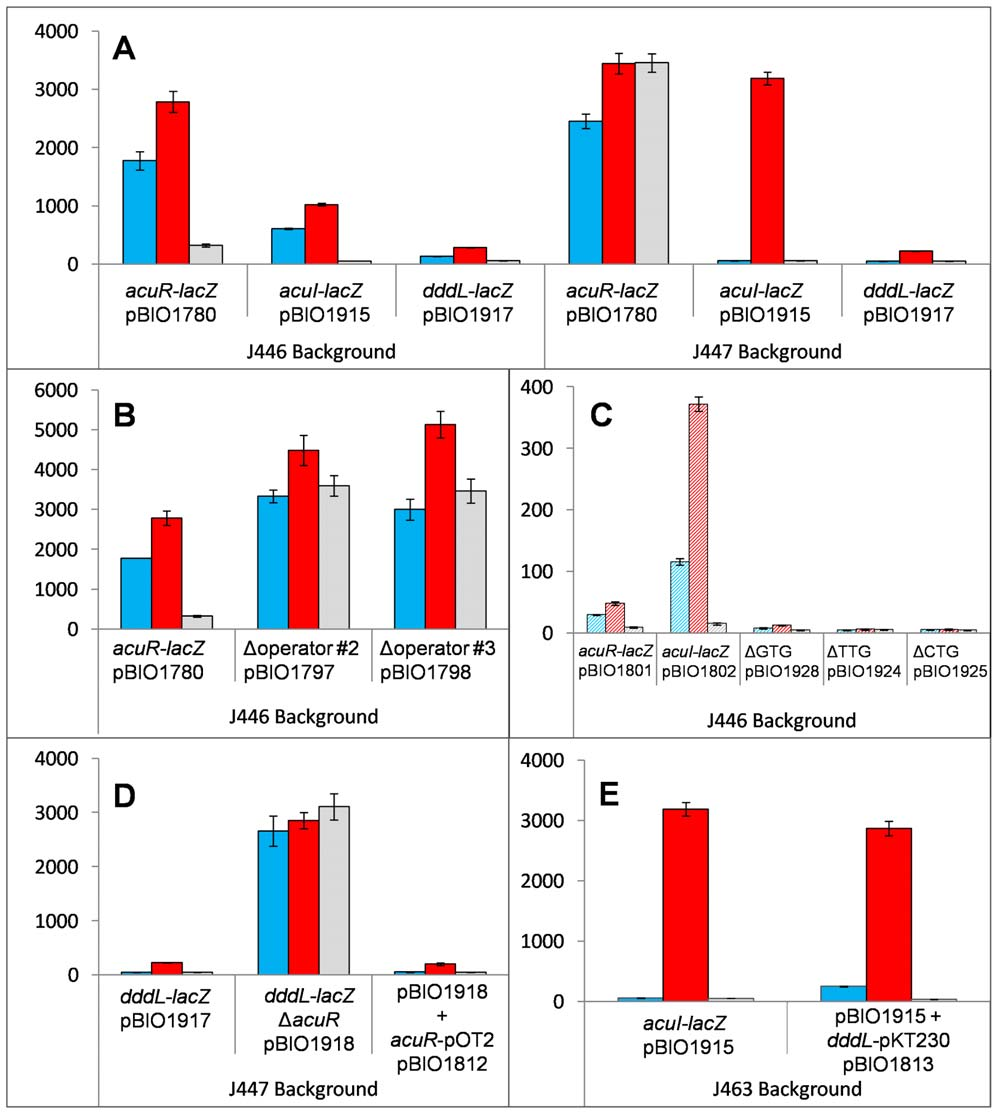
Expression of *acuR-, acuI-* and *dddL-lacZ* transcriptional and translational fusions. The bars show β-galactosidase activities (Miller Units) with standard errors from various *lacZ* fusions to the *acuR, acuI,* or *dddL* genes in either the wild type strain (J446) or in Str^R^ (strain J447) or Rif^R^ (strain J463) derivatives of the “null’ strain 17025 backgrounds, as indicated. Cells were pre-grown in M9 medium supplemented with DMSP (blue), acrylate (red) or no co-inducer (grey). Panels A, B, D, and E show the data for transcriptional fusions using the vector pMP220; panel C has striped bars showing the activity of translational fusions, based on vector pIJ1363. Note that some data for the *acuR-lacZ*, *acuI*-*lacZ* and *dddL-lacZ* fusion plasmids are shown twice for comparisons with the activity of other plasmids, and that the activity values may differ in the Y-axes of different panels. For exact locations and sequences of promoter and operator mutations, see Figure 5.

Each fusion plasmid was transformed into *E. coli* and then conjugated into strain J446, a streptomycin-resistant derivative of *R. sphaeroides* 2.4.1. The transconjugants were assayed for β-galactosidase activity after growth in M9 media, supplemented with succinate as C source, and in the presence or absence of 1 mM DMSP or 1 mM acrylate. All three fusions behaved in a similar way. Compared to the no-inducer control, they all had higher levels (5–19 fold increase) with acrylate, and modest induction (2–10 fold increase) with DMSP (**Figure 3A**). The absolute levels of expression of the genes were greatest for *acuR-lacZ,* followed by *acuI-lacZ,* with the fusion to the promoter-distal gene, *dddL,* being lowest.

The expression of the three genes was also determined by qRT-PCR, as follows. RNA that was harvested from cells pre-grown in M9 minimal medium with or without added acrylate was reverse transcribed and amplified by PCR, using various primer pairs internal to *acuR, acuI* and *dddL*. For all three genes, mRNA levels were greater (∼5–8 fold higher) in cells grown in the presence than in the absence of acrylate (data not shown).

To identify the regulatory gene(s) involved in DMSP- and/or acrylate-dependent expression of the *acuR-acuI-dddL* operon, we first examined the expression of the three fusion plasmids (pBIO1780, pBIO1915 and pBIO1917) in strain J447, a streptomycin-resistant derivative of wild type *R. sphaeroides* ATCC17025, which lacks the *acuR-acuI-dddL* genes and therefore serves as a naturally occurring null mutant for this locus. All three fusions behaved differently compared to when they were in strain J446, which contains these three genes in its genome (**Figure 3A**). Firstly, in strain J447, none of the fusions displayed even the modest induction in response to DMSP. Secondly, although the *dddL-lacZ* fusion (pBIO1917) showed a similar response to acrylate-dependent induction in strain J447 as in strain J446, the *acuI-lacZ* fusion (pBIO1915) was induced more markedly by acrylate when it was in strain J447 (∼60-fold), compared to strain J446 (∼20-fold). With the *acuR-lacZ* fusion, the difference in the expression patterns in the two background strains was even more marked, since in strain J447, *acuR-lacZ* (pBIO1780) was deregulated, with constitutive, high levels of β-galactosidase even in the absence of acrylate.

Taken together, these observations suggest that *acuR, acuI* and *dddL* are co-transcribed in a single operon that can be repressed by AcuR. This would account for the high level constitutive expression in strain J447 harboring the *acuR-lacZ* fusion plasmid pBIO1780, since this strain, unlike any of the others tested, lacks an intact *acuR* gene in the background genome, or the fusion plasmid, or both. The data also indicate that although DMSP appears to relieve the repressive effects of AcuR, it must first be converted to acrylate, the likely *bona fide* co-inducer molecule. This would explain why none of the fusions in the null mutant strain J447 background was inducible by pre-growth in DMSP since these strains lack a functional *dddL* gene that would convert the DMSP to acrylate. The enhanced induction of expression by acrylate of the *acuI-lacZ* fusion in strain J447 compared to the wild type strain J446 background might be because AcuI is involved in acrylate catabolism; if so, the intracellular concentrations of this co-inducer would rise, because strain J447 containing the *acuI-lacZ* fusion plasmid lacks a functional *acuI* gene. These postulates were confirmed directly below.

### The *acuR, acuI* and *dddL* genes are in a leaderless operon

**Figure 4:**
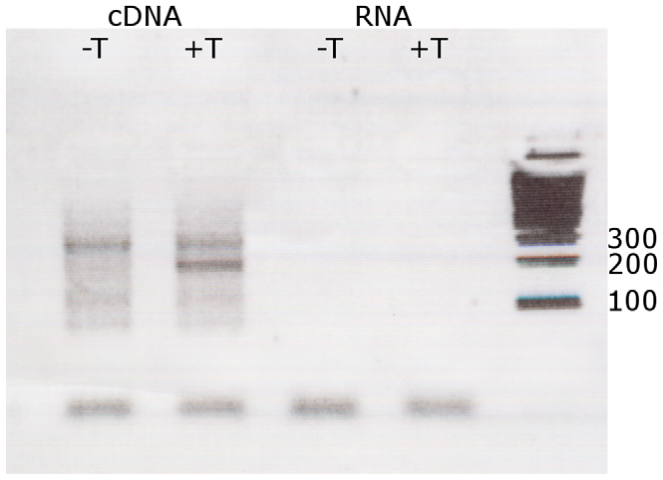
Location of *acuR-acuI-dddL* transcriptional start by 5′RACE RT reactions. Amplification of cDNA from 5′RACE RT reaction, using tobacco acid pyrophosphatase treated (+ T) and untreated (− T) cDNA, or total RNA ligated to RNA adaptor A3. The ∼200 bp product (a) amplified from + TAP-treated cDNA, that is absent from untreated cDNA corresponds to the primary unprocessed mRNA transcript of *acuR* and was the fragment that was sequenced. The cDNA copies (b) of processed RNA products of total-RNA are present in both +T/−T reactions, and are absent from RNA control RT reactions. Size marker values are in base pairs.

**Figure 5:**
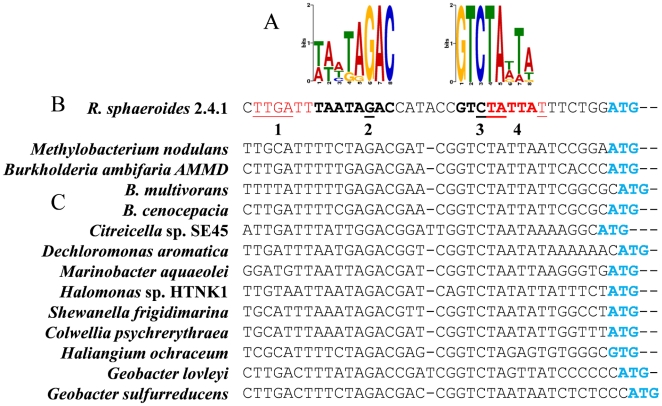
Promoter and operator sequences 5′ of *R. sphaeroides acuR,* compared to corresponding regions in other bacteria. **A.** Sequence logo, using Multiple Em for Motif Elicitation (MEME; see [48]), shows consensus of fourteen sequences of the 8 bp inverted-repeat operator regions upstream of *acuR* in *R. sphaeroides* and of the *acuR-*like genes in the other bacteria shown here.**B.** DNA sequence in the region 36 bp upstream of the ATG (cyan) start codon of *acuR* in *Rhodobacter*. The −10 and −35 motifs of the promoter are in red and the inverted repeats that form the operator in this species are in bold. The locations and identities of the different, plasmid-borne, site-directed mutations in the *R. sphaeroides* promoter and operator are underlined, and numbered as follows: 1. TTGATT to **CGAG**TT promoter mutation, used in plasmids pBIO1829 and pBIO1923. 2. G to **C** operator mutation, used in plasmid pBIO1797. 3. C to **G** operator mutation, used in plasmid pBIO1798. 4. TATTAT to **GC**TTA**C** promoter mutation, used in plasmids pBIO1827 and pBIO1922. **C**. Sequences of corresponding regions upstream of the *acuR-*like genes in other bacteria.

The transcriptional start of the *acuR-acuI-dddL* operon was identified by 5′ **R**apid **A**mplification of **c**DNA **E**nds (RACE), using total RNA isolated from cells of strain 2.4.1 grown in LB supplemented with 1 mM acrylate. Primary mRNA transcripts were distinguished using tobacco alkaline pyrophosphatase (TAP), and primers internal to *acuR* were used to reverse transcribe and amplify *acuR-acuI-dddL* mRNA specifically, prior to cloning cDNA copies into pGEM-T-Easy (Materials and Methods). Sequencing 10 independent, cloned copies of the *acuR* primary transcript (**Figure 4**) showed that it began exactly at the *acuR* 5′-AUG start codon. Such a leaderless transcript is unusual in Gram negative bacteria, but is fully consistent with the fact that we found no convincing Shine-Dalgarno (SD) site immediately 5′ of the predicted *acuR* ATG start codon (**Figure 5B**). This ATG is highly conserved in AcuR homologues in other bacteria and was shown directly to be required for the translation of the AcuR polypeptide (see below).

Centred 10 and 35 bp upstream of this +1 transcriptional start were TATTAT and TTGATT motifs, potential RNA polymerase recognition sites (**Figure 5B**). To confirm that these formed the *acuR-acuI-dddL* promoter, they were subjected to site-directed mutagenesis (SDM) as follows. The fragments that were used to make the *acuR-lacZ* fusion pBIO1780, and the *dddL-lacZ* fusion pBIO1917, were each cloned into pBluescript [27]. Conserved nucleotides (labelled “1” and “4” in **Figure 5B**) in these −10 and −35 motifs were then mutated and the mutated fragments were individually re-cloned into pMP220. The resulting plasmids (pBIO1827, pBIO1829, pBIO1922 and pBIO1923) were each mobilised into the *R. sphaeroides* strains J446 and J447. None of these mutated fusions expressed β-galactosidase above background in either host strain, confirming the importance of these −10 and −35 motifs for promoter activity (data not shown). Furthermore, the fact that mutations in the promoter upstream of *acuR* abolished expression of the downstream *dddL* gene confirms that *acuR, acuI* and *dddL* are co-transcribed. Further evidence for the lack of any internal promoters came from the finding that two transcriptional fusion plasmids, containing the intergenic spaces between *acuR* and *acuI* (pBIO1784) or between *acuI* and *dddL* (pBIO1785) cloned into pMP220 (**Figure 2**), had no detectable β-galactosidase activity in *R. sphaeroides* strains J446 or J447 that had been grown in the presence or absence of DMSP or of acrylate (data not shown).

Overlapping the *acuR-acuI-dddL* promoter is an inverted repeat, TAATAGAC/n6/GTCTATTA, which is a potential *cis-*acting regulatory operator (**Figure 5A**). To confirm this, we mutated these motifs (marked “2” and “3” in **Figure 5B**) by site-directed mutagenesis of the *acuR-lacZ* fusion plasmid (pBIO1780), altering the most highly conserved bases, but avoiding those that coincided with the promoter (**Figure 5**). The resultant mutant plasmids (pBIO1797, pBIO1798) were conjugated into *R. sphaeroides* before assaying β-galactosidase after growth in DMSP or acrylate or with no inducer. Both sets of mutations caused high-level, constitutive expression (**Figure 3B**) when compared to the un-mutated *acuR-lacZ* fusion, consistent with these being in the *cis*-acting operator site to which the AcuR repressor binds.

### Lack of ribosomal binding site diminishes expression of *acuR*

The mRNA of the *acuR-acuI-dddL* operon lacked a 5′ leader and so *acuR* lacks a SD site, whereas both *acuI* and *dddL* have such motifs (AGGAGA and AGAGAG, respectively) upstream of their AUG start codons. Thus, there might be differences in the translational efficiencies of the genes in the operon. To investigate this, we made two *lacZ* translational fusions in the wide host-range cloning vector pIJ1363 [28], whose *lacZ* gene lacks both a promoter and a SD site. In these fusions, fragments that spanned the *acuR-acuI-dddL* promoter and operator were cloned into pIJ1363 (**Figure 2**) to form translational fusion plasmids pBIO1801 (*acuR-lacZ*) and pBIO1802 (*acuI-lacZ*). These two plasmids were conjugated into wild type *R. sphaeroides* strain J446. Transconjugants were grown with or without acrylate, and assayed for β-galactosidase (**Figure 3C**). Strikingly, in the acrylate-grown cells, the *acuR-lacZ* (pBIO1801) translational fusion was expressed at around ∼7.5% of the levels observed with the *acuI*-*lacZ* translational fusion (pBIO1802), even though the *lacZ* transcriptional fusions and qRT-PCR (above) showed that *acuR* was transcribed at higher levels than *acuI*.

To confirm the importance of the ATG start codon for the expression of the *acuR-lacZ* translational fusion pBIO1801, we mutated this to three alternative versions, namely TTG, CTG, and GTG (pBIO1924, pBIO1925 and pBIO1928, respectively) and mobilised the resultant mutant fusion plasmids into strain J446. All but one of the mutated plasmids expressed no detectable β-galactosidase activity; the exception was where the ATG was converted to the usually less efficient start codon GTG (pBIO1928), in which the expression of the translational fusion was much-reduced, but not abolished (**Figure 3C**).

### AcuR is an acrylate-responsive repressor of *acuR-acuI-dddL*

To show directly that AcuR represses *acuR-acuI-dddL*, we made a derivative of the *dddL-lacZ* fusion plasmid pBIO1917 with an in-frame deletion in *acuR* (558 of 660 bp removed), forming pBIO1918 (**Figure 2**). This plasmid was mobilised into the “null” strain J447 and transconjugants were assayed for β-galactosidase after growth in the presence or absence of DMSP or acrylate. This *acuR* deletant fusion plasmid showed high-level, constitutive β-galactosidase activity on all three media, consistent with the regulatory model set out above (**Figure 3D**). This defect in regulation was fully restored by introducing pBIO1812, in which *acuR* had been cloned in the wide host-range vector pOT2 [29]. Thus AcuR, supplied *in trans,* is a transcriptional repressor of the *acuR-acuI-dddL* operon.

### Induction of *acuR-acuI-dddL* by DMSP requires its transformation to acrylate via *dddL*

To confirm that the apparent induction of expression of the *acuR-acuI-dddL* operon requires the substrate DMSP to be converted to acrylate, the *dddL* gene was cloned into the wide host-range vector pKT230 [30], under the control of its *str* promoter, to form pBIO1813. When this plasmid was mobilised into J463 (a Rif^R^ mutant of the null strain ATCC17025) containing the *acuI-lacZ* fusion plasmid pBIO1915, this restored the ability of DMSP to co-induce this fusion (**Figure 3E**), consistent with this model.

### The role of AcuI in acrylate catabolism

Acrylate is produced by DddL-mediated catabolism of DMSP and it is also a co-inducer of *acuR-acuI-dddL*, so we examined the possible role of this operon in acrylate catabolism. In particular, we investigated the product of the *acuI* gene, a predicted dehydrogenase, due to its similarity (21% identical) to 2-haloacrylate reductase of *Burkholderia* sp. WS [31], another member of the Zn-dependent oxido-reductases that reduces 2-chloroacrylate to 2-chloropropionate.

**Figure 6:**
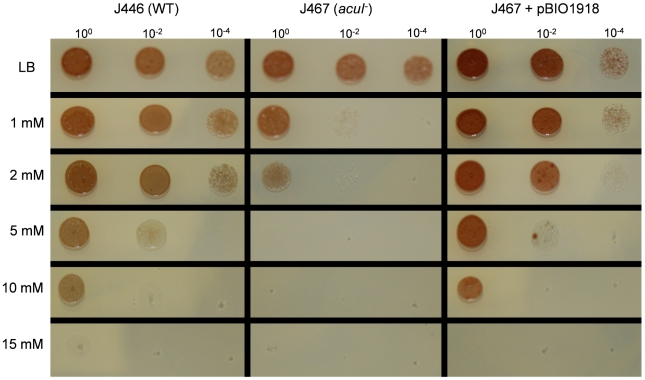
Effect of acrylate on the viability of wild type and AcuI^−^ mutant *R. sphaeroides*. Cultures of *R. sphaeroides* wild type strain J446, the AcuI^−^ mutant strain J467, and strain J467 corrected with cloned *acuI* (pBIO1918) were diluted as indicated and 10 µl spots placed on LB media lacking acrylate or with increasing concentrations of acrylate as shown. The plates were incubated at 28°C for 36 hours.

An insertional mutation in the genomic copy of *acuI* in *R. sphaeroides* strain J446, forming mutant strain J467, was made as described in Materials and Methods. Since wild type *R. sphaeroides* itself does not grow on acrylate as sole C source, it was not possible to test if the mutation affected this ability. However, we had noted that >10 mM acrylate in the medium inhibited the growth of *R. sphaeroides* strain 2.4.1 and we investigated this phenotype. The AcuI^−^ mutant was significantly more sensitive to acrylate than the wild type parent and we showed that this hypersensitivity was overcome by reintroducing pBIO1918, a plasmid that expresses functional *acuI* (**Figure 6**). Thus, AcuI is involved, directly or indirectly, in a process that counteracts the toxic effects of acrylate. Compared to its sensitivity to acrylate, *R. sphaeroides* is relatively resistant to DMSP, and no difference was seen in the tolerance of the wild type and the AcuI^−^ mutant, up to a concentration of 50 mM DMSP in the growth medium.

**Figure 7:**
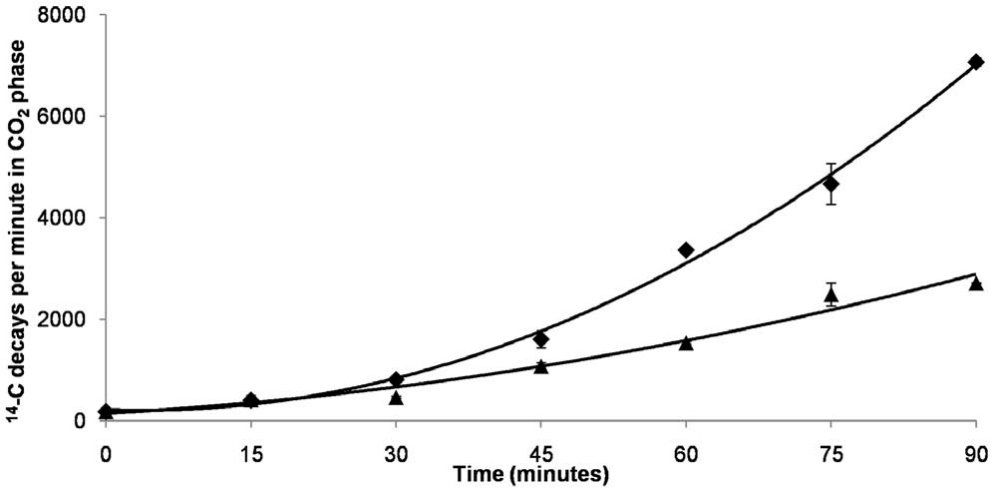
Effect of an *acuI* mutation on the production of ^14^CO_2_ from [1−^14^C] acrylate by *R. sphaeroides*. Cultures of wild type strain J446 (diamonds) and AcuI^−^ mutant J467 (triangles) were assayed for ^14^CO_2_ production as described in Experimental Procedures. Results are averaged from 2 replicate experiments and standard errors are <±5%.

To examine more directly if AcuI was involved in acrylate catabolism, we fed [1−^14^C] acrylate to cultures of wild type and AcuI^−^ mutant strains of *R. sphaeroides* 2.4.1, in which the *acuR-acuI-dddL* operon had been induced by pre-growth of the cells in the presence of acrylate. The disappearance of labelled substrate and the appearance of soluble and gaseous catabolite(s) in the cells and in the extracellular medium were then monitored. Both strains removed the labelled acrylate, and produced concomitant amounts of labelled ^14^CO_2_, but the rates for both parameters were significantly greater in the wild type than in the AcuI^−^ mutant (**Figure 7**). However, under these conditions no labelled compounds were seen that might correspond to intermediates in the acrylate catabolic pathway.

### *acuR*- and *acuI*-like genes in other bacteria

Interestingly, the closest homologues (≥50% identical) of the products of both *acuR* and of *acuI* were also encoded by adjacent genes in the genomes of several individual strains of α-, β- and γ-Proteobacteria and one *Verrucomicrobia* (**Figure 1**). In all but one of these cases, the particular substrate of the AcuI-like enzyme and the co-inducer for the AcuR-like regulators are unknown and/or have no known link to DMSP catabolism. The exception is the gene pair in *Halomonas* HTNK1, which are closely linked to the previously described *ddd* and *acu* genes involved in the catabolism of DMSP and acrylate [19]. We also noted that the Global Ocean Sampling data set [32] of marine bacterial metagenomes has six close matches (<e^−40^) to AcuR, four of which were encoded by genes that were next to *acuI-*like genes. Thus, this pair-wise arrangement of adjacent genes related to *acuI* and *acuR* is widespread in the genomes of different bacteria.

We noted that the DNA immediately 5′ of several of the *acuR-*like genes in other bacteria that had adjacent *acuR-*like/*acuI-*like genes (**Figure 1**), also contained motifs that resemble those in the operator/promoter of the *R. sphaeroides acuR-acuI-dddL* operon. Furthermore, their mRNAs are predicted to lack 5′ leader sequences or SD sites (**Figure 5C**).

We directly examined the expression of one such gene pair, in the β-Proteobacterium, *Burkholderia ambifaria* AMMD, which contains the *acuR-*like gene Bamb_4743 (58% identical to AcuR at the gene product level) and Bamb_4744 (61% identical to AcuI and separated from Bamb_4743 by 46 bp). We made two transcriptional fusion plasmids in pMP220, both of which contained the predicted promoter and operator of this *B. ambifaria* operon. The 3′ end of one of these (pBIO1807) was in Bamb_4743 and, in the other (pBIO1808), the 3′ terminus was in Bamb_4744. These two fusion plasmids were mobilised into *R. sphaeroides* strain J446. The Bamb_4743*-lacZ* transcriptional fusion was expressed at high levels (∼300 Miller units of β-galactosidase), both in the presence or absence of DMSP or acrylate; thus, its promoter was not repressed by AcuR in strain 2.4.1. However, the *acuI*-like Bamb_4744*-lacZ* fusion was expressed at lower (∼4 Units) levels in both conditions, suggesting that the product of Bamb_4743 (which is intact in the pBIO1808 fusion plasmid, but not in pBIO1807) represses its own transcription, but that it does not respond to acrylate or to DMSP.

## Discussion

This work reveals two novel features of the organisation and regulation of the *dddL-*containing operon of *R. sphaeroides -* (i) unusually for Proteobacteria, its mRNA product lacks an untranslated leader, with no SD sequence and (ii) it is induced primarily in response to the catabolite acrylate and only indirectly by the substrate DMSP.

The mRNA of the *acuR-acuI-dddL* operon lacks an untranslated 5′ leader. Conventionally, bacterial mRNAs contain a Shine-Dalgarno (SD) sequence immediately 5′ of the translational start codon, which is involved in recruiting ribosomes to initiate translation. However, a few bacterial genes lack such upstream untranslated leaders, as first shown for the *cI* repressor of bacteriophage λ [33], then the *tetR* gene of transposon Tn*1721* [34]. Other cases have since been described or inferred by bioinformatic analyses and some understanding on how such leaderless messages are translated has emerged [35]. Leaderless mRNA templates are recognised by ribosomes, using fMet-tRNA_f_^Met^ initiator tRNA plus initiation factor IF2, in a mechanism that resembles that in eukaryotes, which also have no specialised ribosomal binding sites upstream of the initiation codon. This may reflect the ancestral form of translational initiation of such leaderless transcripts [36].

In Proteobacteria, several genes with ratified leaderless transcripts encode regulatory proteins (e.g. λ CI and *E. coli* TetR), which are not required in large amounts, consistent with the reduced levels of translation of genes that lack ribosomal binding sites, compared to those that do [37]. Leaderless transcripts are more frequent in some Gram positive bacteria and in Archaea, in which a wider range of functions are encoded by the corresponding genes [35]. And more recently, genome-wide surveys have shown that leaderless transcripts may be more widespread than previously thought; in *Helicobacter pylori*, for example, ∼2% of the transcripts that were identified had very short (<10 bp) 5′ extensions and in 26 cases, the 5′ end of the mRNA was located exactly at the AUG translational start [38], as found here with *acuR*.

By comparing the responses of transcriptional and translational fusions, the work on *acuR-acuI-dddL* is one of the few direct demonstrations that an upstream regulatory gene (*acuR*) in a leaderless mRNA transcript is transcribed at least as efficiently as the downstream genes, but is translated at far lower levels. This type of gene control may also apply to the other cases where an *acuR*-like gene lies upstream of an *acuI-*like gene, an arrangement that we found in a taxonomically wide range of known bacteria as well as in sub-genomic fragments of the metagenomes of marine bacteria. In all such cases, the region immediately 5′ of the *acuR* homologue lacked a SD site, and had convincing -10 and -35 promoter motifs located such that the transcript would start at the AUG of the promoter-proximal mRNA. Further, many of these *acuR-acuI*-like gene pairs had regulatory sequences similar to those in the *acuR-acuI-dddL* operator of *R. sphaeroides.*

Acrylate, the product of DMSP catabolism is the co-inducer for *acuR-acuI-dddL* expression. The finding that a *product* (acrylate) of a catabolic reaction appears to be the primary co-inducer of the corresponding gene (*dddL*) is unusual, but not unprecedented in bacteria. This is not the only case in which DMSP catabolism is induced by a catabolite, Yoch's laboratory having described several instances in which pre-growth of different bacteria in the presence of acrylate or of 3HP enhances their rates of DMSP-dependent DMS production (see [5]).

In the case of *acuR-acuI-dddL*, a possible reason for this mode of regulation stems from the way in which this operon may have evolved, as follows. So far, the *dddL* gene is confined to a relatively small number of bacterial species, nearly all in the Rhodobacteraceae family of α-Proteobacteria. In all but one of these species, *dddL* is in a one-gene transcriptional unit, the exception being *R. sphaeroides.* Perhaps by HGT, a *dddL* gene “latched onto” a pre-existing *R. sphaeroides* operon that comprised adjacent *acuR*-*acuI-*like genes, whose role may have been involved in catabolising acrylate (or a similar molecule) and whose expression therefore, responded to acrylate. Little is known of the distribution of acrylate in natural environments, but it does occur in regions of high DMSP production, such as coral reefs where it is made by cleavage of DMSP [39]. Therefore, acrylate could act as a surrogate to activate genes involved in DMSP catabolism, since, where acrylate occurs, DMSP may also be found [40].

Although genetic analyses of DMSP catabolism have only been ongoing for a few years, these studies have already uncovered a remarkable diversity in different aspects of this process, from the types of enzymes involved in the initial biotransformation, to the range of microbial lineages that can accomplish it. The work presented here now reveals novelty and diversity in the regulation of this process, since the acrylate-dependent expression via a TetR-type regulator in *Rhodobacter* contrasts markedly with that in the γ-Proteobacterium *Marinomonas,* where a LysR-type regulator responds to DMSP itself [17]. It will be of interest to see if yet other mechanisms regulate the catabolism of this abundant and important molecule in other microbes.

## Materials and Methods

### Bacterial strains, plasmids and media

Strains and plasmids are listed in supplementary table S1. *E. coli* and *R. sphaeroides* were grown at 37°C and 28°C respectively on Luria-Bertani (LB) or M9 minimal media [41]. Antibiotics were used at the following concentrations (µg ml^−1^): Str (400), Kan (20), Tet (5 for *E. coli*, 1 for *R. sphaeroides*), Amp (100), Gem (20 for *E. coli*, 80 for *R. sphaeroides*) and Rif (20).

To assay β-galactosidase, cells pre-grown in LB were diluted 10^−2^ in M9 media with 10 mM succinate as C source, with or without 1 mM DMSP or acrylate, and incubated overnight, prior to being assayed as in Rossen *et al*. [28].

### *In vitro* and *in vivo* genetic manipulations

General handling and manipulation of DNA were done as in [42]. Plasmids were conjugated from *E. coli* to strains of *Rhodobacter sphaeroides* by triparental mating using helper plasmid pRK2013 [43].

### Gene amplification and construction of plasmids and mutants

Fragments of *R. sphaeroides* genomic DNA were amplified by PCR using primers that contained appropriate restriction sites, listed in supplementary table S2. Recombinant plasmids based on pBluescript were transformed into *E. coli* strain JM101 [44], and for plasmids based on larger vectors (pOT2, pKT230, pIJ1363 and pMP220) the recipient strain was *E. coli* 803 [45]. The dimensions and names of the relevant plasmids are shown in **Figure 2** and described in supplementary table S1.

An in-frame deletion of >85% of the *acuR* gene was made using a QuikChange Lightning mutagenic PCR kit according to the manufacturer's instructions (*Agilent*). The template was the 2.2 kB *Eco*RI-*Pst*I fragment from pBIO1917 sub-cloned into pBluescript and the primer pairs are in supplementary table S2. The products were transformed into *E. coli* XL10-Gold Ultracompetent cells (*Agilent*), selecting ampicillin resistance. Mutant plasmids were ratified by sequencing, and then the mutagenised insert was sub-cloned back into the wide host-range plasmid pMP220 to form pBIO1918, which was sequenced for verification of the mutation.

Base-pair substitutions in the promoter and operator regions 5′ of *acuR* were made using the QuikChange Lightning mutagenic PCR kit according to the manufacturer's instructions (*Agilent*). The templates were the ∼600 bp *Eco*RI-*Pst*I fragment from pBIO1780 sub-cloned into pBluescript, and the 2.2 kB *Eco*RI-*Pst*I fragment from pBIO1917 sub-cloned into pBluescript. The mutagenic primer pairs are shown in supplementary table S2.

### Construction of plasmid integration mutant

A 618 bp fragment, internal to *acuI*, was amplified from *R. sphaeroides* 2.4.1 genomic DNA using forward and reverse primers (shown in supplementary table S2) which respectively contain *Eco*RI and *Pst*I restriction sites. This *Eco*RI-*Pst*I fragment was cloned into the suicide plasmid vector pK19*mob* [46] to make pBIO1831. This plasmid was then conjugated to *R. sphaeroides* strain J446 (as above) and recombined into the genomic *acuI* region via a single crossover, selected by resistance to streptomycin (strain J446) and kanamycin (pK19*mob*), and confirmed by colony PCR. This AcuI^−^ integration mutant was designated strain J467.

### Assays for DMS production

Cultures of *R. sphaeroides* were diluted 10^−2^ into 5 ml M9 medium containing 10 mM succinate, with or without 1 mM DMSP, or acrylate. After overnight incubation, the cells were washed, resuspended in 300 µl M9 medium containing 10 mM succinate and 5 mM DMSP substrate adjusted to pH 6.5, and placed in a sealed vial (12×32 mm, *Alltech Associates*). DMS in the headspace was assayed by gas chromatography at intervals as in [19]. Protein concentrations were estimated using Bradford assays (*BioRad*).

### RNA extraction and quantitative real-time RT-PCR

Starter cultures of *R. sphaeroides* strain 2.4.1 were grown overnight in LB media, washed and diluted 10^−2^ in M9 minimal media containing 10 mM succinate and 1 mM acrylate, and incubated at 28°C for 14 hours to an OD_600_ of 0.4. Two 5 ml aliquots of each culture were harvested and added to 0.4 culture volumes of ice-cold 5% phenol, 95% ethanol (v/v) solution, and incubated on ice for 1 hour to stabilise RNA and prevent degradation. Cells were then pelleted and RNA was extracted using SV Total RNA isolation kit (*Promega*). The absence of genomic DNA contamination was confirmed by PCR amplification of RNA samples. For qRT-PCR, primers (see supplementary Table 2) were used to amplify *acuR*, *acuI* and *dddL* from total RNA isolated from cells grown in the presence or absence of acrylate. Normalisation of mRNA levels was performed using the *rpoZ* gene. The iScript™ One-step RT-PCR Kit with SYBR® Green (*BioRad*) was used for reverse transcription followed by PCR as in the manufacturer's manual. Master mix and RNA solutions were added to a final volume of 25 µl, containing 50 ng RNA, and mRNA transcripts were quantified using the C1000 Thermal cycler and CFX96 Real-Time PCR detection system (*BioRad*).

### Mapping 5′ transcriptional start site and promoter of *acuR*

5′-RACE assays were carried out essentially as described by Bensing *et al.* [47] with some modifications. The 5′-triphosphates of 6 µg total-RNA were converted to monophosphates with 10 units of TAP (*Epicentre Technologies*) according to manufacturer's specifications. Control RNA was incubated under the same conditions in the absence of the enzyme. 500 pmol RNA-adapter oligo A3 was ligated to total primary transcripts using T4 RNA ligase (*New England Biolabs*) according to manufacturer's specifications. ThermoScript Reverse Transcriptase (*Invitrogen*) was used with 2 pmol of acuR_5RACE1 primer to reverse transcribe the ligated *acuR-acuI-dddL* transcripts into cDNA. HotStarTaq DNA polymerase (*QIAGEN*) and 25 pmol of DNA_oligo_B6 and acuR_5RACE2 primers were used to amplify cDNA copies of the *acuR-acuI-dddL* transcript. RNA ligation reactions were used as control templates to indicate the absence of DNA contamination. A single band corresponding to DNA copies of the TAP-treated *acuR-acuI-dddL* primary transcript was gel extracted using the QIAEX II Gel extraction kit (*QIAGEN*), cloned into pGEM-T-Easy vector system (*Promega*) and transformed into *E. coli* DH5α. Colony PCR using M13F and M13R primers was used to confirm cloning success, and 10 individual plasmids were sequenced using the AbiPrism 3730 capillary sequencer (The Genome Analysis Centre, Norwich Research Park, United Kingdom).

### Assays for ^14^CO_2_ generated from [1−^14−^C] acrylate

Measurement of ^14^CO_2_ production from [1−^14−^C] acrylate was done essentially as described by [19]. *R. sphaeroides* strains J446 and J467 were grown overnight in complete media supplemented with 1 mM acrylate, adjusted to equivalent OD_600_ values of 0.8, washed three times and resuspended in 1 ml M9 minimal media containing 10 mM succinate, in 25 ml *Sterilin* universal tubes. Within each tube was fastened an open 1.5 ml microfuge tube containing 500 µl 10 M KOH, to capture the ^14^CO_2_. Then 1.9 kBq [1−^14−^C] acrylate was added to each culture, together with unlabelled acrylate as appropriate to a final concentration of 1 mM, before immediately sealing the *Sterilin* tube, which was then incubated at 28°C. Radioactivity was measured separately in the KOH trap and in the culture supernatant. Each sample was mixed with ULTIMA-FLO AP scintillation fluid, before counting in a Wallac 1409 scintillation counter (*Perkin Elmer*). Replicate cultures were used to measure ^14^CO_2_ production at 15 minute intervals, for 90 minutes.

### Identification of [1−^14−^C] acrylate metabolites by HPLC

Cultures of *R. sphaeroides* strain J446 were exposed to [1−^14−^C] acrylate as described above. Following incubation at 28°C for set time periods, cells were lysed by adding 5% (v/v) perchloric acid and incubated on ice for 10 minutes. Cell debris was pelleted and 0.1 ml supernatant was added to HPLC vials. Acrylate and other metabolites were resolved on an ICE-AS6 column (250 mm×9 mm id., *Dionex*), essentially as described in Todd *et al.* [19].

### Acrylate toxicity experiments

Starter cultures of strain J446, the AcuI^−^ mutant strain J467 and strain J467 containing the plasmid pBIO1918 were grown overnight in LB. Cells were adjusted to equivalent OD_600_ values, washed and serially diluted, and 10 µl spots of the 10^−4^, 10^−2^ and undiluted cultures were plated on LB agar supplemented with increasing levels (1 mM to 15 mM) acrylate. Plates were then incubated at 28°C for 36 hours.

## Supporting Information

Table S1 Strains and Plasmids.
(DOCX)

Table S2 Sequences that are underlined show the mutated bases and those in bold indicate restriction sites used for cloning.
(DOC)
