# Supplementary material for: Unusual Regulation of a Leaderless Operon Involved in the Catabolism of Dimethylsulfoniopropionate in Rhodobacter sphaeroides
Source: PLoS One. 2011 Jan 7;6(1):e15972. doi: 10.1371/journal.pone.0015972 (PMC3017554; doi:10.1371/journal.pone.0015972)
Supplement: Table S1 — Strains and Plasmids. (DOCX) [file pone.0015972.s001.docx]

**Supplementary Table S1**

**Strains and Plasmids**

| **Bacteria** | **Characteristics** | **Source** |
| --- | --- | --- |
| *Escherichia coli* 803 | Met^-^; used as host for transformation with large plasmids | [45] |
| *E. coli* JM101 | Used as host for pUC18-based plasmids | [44] |
| *E. coli* DH5α | Used for transformation of pGEM-T-Easy-based plasmids | *Bethesda Research Laboratories* |
| *E. coli* XL10-Gold | Used for the transformation of mutagenised products | *Stratagene* |
| *Rhodobacter sphaeroides* 2.4.1 | Wild type Ddd^+^ strain;  http://genome.jgi-psf.org/rhosp/rhosp.home.html | [24] |
| *R. sphaeroides* ATCC17025 | Wild type Ddd^-^ strain; <http://genome.jgi-psf.org/draft_microbes/rhos5/rhos5.home.html> | [24] |
| *R. sphaeroides* J446 | Str^R^ mutant of *R. sphaeroides* 2.4.1 | This work |
| *R.* *sphaeroides* J447 | Str^R^ mutant of *R. sphaeroides* ATCC17025 | This work |
| *R. sphaeroides* J463 | Rif^R^ mutant of *R. sphaeroides* ATCC 17025 | This work |
| *R. sphaeroides* J467 | AcuI^-^ mutant derivative of J446, with suicide plasmid pBIO1831 inserted in *acuI* (Str^R^ Kan^R^) | This work |
| *Loktanella vestfoldensis* SKA53 | Wild type Ddd^+^ strain | http://www.moore.org/microgenome/detail.aspx?id=38 |
| *Fulvimarina pelagi*  HTCC2506 | Wild type Ddd^+^ strain | http://www.moore.org/microgenome/detail.aspx?id=31 |
| *Sulfitobacter* EE-36 | Wild type Ddd^+^ strain | http://www.moore.org/microgenome/detail.aspx?id=41 |
| *Stappia aggregata*  IAM 12614 | Wild type Ddd^+^ strain | http://www.moore.org/microgenome/detail.aspx?id=130 |
| *Burkholderia ambifaria* AMMD | Wild type Ddd^+^ strain | http://genome.jgi-psf.org/buram/buram.home.html |
| **Plasmids** | **Characteristics** | **Source** |
| pRK2013 | Used as mobilising plasmid in tri-parental crosses (Kan^R^) | [43] |
| pMP220 | Wide host-range promoterless-*lacZ* probe vector (Tet^R^) | [26] |
| pKT230 | Wide host-range expression vector (Str^R^, Kan^R^) | [30] |
| pIJ1363 | Broad host-range translational *lacZ*-protein fusion vector (Tet^R^) | [28] |
| pGEM-T Easy | Used for cloning and sequencing PCR products from 5´RACE PCR amplification | *Promega* |
| pOT2 | Broad host-range promoter probe vector with promoterless *gfp* gene (Gem^R^) | [29] |
| pBluescript M13 | M13 phagemid T_7_ T_3_ *lacI* *lacZ* (Amp^R^) | [27] |
| pK19mob | Suicide insertion plasmid, (Kan^R^) | [46] |
| pBIO1780 | pMP220-based transcriptional *acuR-lacZ* fusion plasmid, (Tet^R^) | This work |
| pBIO1784 | pMP220-based transcriptional fusion plasmid spanning the intergenic space between *acuR* and *acuI*, (Tet^R^) | This work |
| pBIO1785 | pMP220-based transcriptional fusion plasmid spanning the intergenic space between *acuI* and *dddL*, (Tet^R^) | This work |
| pBIO1797 | pMP220-based *acuR-lacZ* transcriptional fusion plasmid, with G to C transversion in operator, 24 bp upstream of *acuR,* (Tet^R^) | This work |
| pBIO1798 | pMP220-based *acuR-lacZ* transcriptional fusion plasmid, with C to G transversion in operator, 13 bp upstream of *acuR*, (Tet^R^) | This work |
| pBIO1801 | pIJ1363-based *acuR-lacZ* translational fusion, (Tet^R^) | This work |
| pBIO1802 | pIJ1363-based *acuI-lacZ* translational fusion, (Tet^R^) | This work |
| pBIO1807 | pMP220-based transcriptional-*lacZ* fusion to *Burkholderia acuR-like* gene Bamb_4743, (Tet^R^) | This work |
| pBIO1808 | pMP220-based transcriptional-*lacZ* fusion to *Burkholderia acuI-like* gene Bamb_4744, (Tet^R^) | This work |
| pBIO1812 | pOT2 derivative containing *acuR* and its upstream promoter region, (Gem^R^) | This work |
| pBIO1813 | pKT230 derivative containing *dddL* and its ribosome binding site, (Str^r^  Kan^R^) | This work |
| pBIO1827 | pBIO1780 derivative with 3 bp substitution in -10 motif of *acuR* promoter, (Tet^R^) | This work |
| pBIO1829 | pBIO1780 derivative with 4 bp substitution in -35 motif of *acuR* promoter, (Tet^R^) | This work |
| pBIO1831 | pK19mob derivative with a 618 bp fragment internal to *acuI*, (Kan^R^) | This work |
| pBIO1915 | pMP220-based transcriptional *acuI-lacZ* fusion plasmid, (Tet^R^) | This work |
| pBIO1917 | pMP220-based transcriptional *dddL-lacZ* fusion plasmid, (Tet^R^) | This work |
| pBIO1918 | AcuR*^-^* deletant derivative of pBIO1917, (Tet^R^) | This work |
| pBIO1922 | pBIO1917 derivative, with 3 bp substitution in -10 motif of *acuR* promoter, (Tet^R^) | This work |
| pBIO1923 | pBIO1917 derivative, with 4 bp substitution in -35 motif of *acuR* promoter, (Tet^R^) | This work |
| pBIO1924 | pBIO1801 derivative, with altered *acuR* start codon to TTG, (Tet^R^) | This work |
| pBIO1925 | pBIO1801 derivative, with *acuR* start codon altered to CTG, (Tet^R^) | This work |
| pBIO1928 | pBIO1801 derivative, with *acuR* start codon altered to GTG, (Tet^R^) | This work |
