# Supplementary material for: Unusual Regulation of a Leaderless Operon Involved in the Catabolism of Dimethylsulfoniopropionate in Rhodobacter sphaeroides
Source: PLoS One. 2011 Jan 7;6(1):e15972. doi: 10.1371/journal.pone.0015972 (PMC3017554; doi:10.1371/journal.pone.0015972)
Supplement: Table S2 — Sequences that are underlined show the mutated bases and those in bold indicate restriction sites used for cloning. (DOC) [file pone.0015972.s002.doc]

**Supplementary Table S**2

| **Primer** | **Sequence** | **Used for** |
| --- | --- | --- |
| acuRPstIR1 | GCTCT**CTGCAG**GCCTGCGCCTCCCG | For cloning fragment used in pBIO1780 |
| acuREcoRIF1 | CCAAGTCA**GAATTC**GCGTGTTTCCCG | For cloning fragment used in pBIO1780, pBIO1915, pBIO1917 |
| acuRIgapF1 | GGCCCGCCAC**GAATTC**CGGCGCGG | For cloning fragment used in pBIO1784 |
| acuRIgapR1 | CGCAACG**CTGCAG**ACACCGCC | For cloning fragment used in pBIO1784, pBIO1915 |
| acuILgapF1 | GCAAC**GAATTC**ACCGGCAAGGTCC | For cloning fragment used in pBIO1785 |
| acuILgapR1 | GC**CTGCAG**CACGGGCCAGCGCCCTCG | For cloning fragment used in pBIO1785 |
| acuR_XbaIFor | ccaa**tctaga**acccgcgtgtttcccg | For cloning fragment used in pBIO1812 |
| acuR_PstIrev1 | cggatttctc**ctgcag**aacggc | For cloning fragment used in pBIO1812 |
| 241dddLEcoFOR3 | GGACGCATCG**GAATTC**CGGTCACCCCCTGACCTGC | For cloning fragment used in pBIO1813 |
| dddL_EcoRIr1 | CCCGTCG**GAATTC**ACGCAGCGGC | For cloning fragment used in pBIO1813 |
| acuRIdddL_BamHfor1 | CCCGCGT**GGATCC**CGACCTCGCGCTGC | For cloning fragment used in pBIO1804, pBIO1805, pBIO1806 |
| acuR_BamHIrev1 | CGAGGAAAGCC**GGATCC**AGAAGCCTGGC | For cloning fragment used in pBIO1804 |
| acuI_BamHIrev1 | CGACGCGGGC**GGATCC**ATTCCCGCGGC | For cloning fragment used in pBIO1805 |
| dddL_BamHIrev1 | CAACGCCAAG**GGATCC**CGCACCCGCTT | For cloning fragment used in pBIO1806 |
| TR_G-C_SDMFp1 | CGCACTTGATTTAATACACCATACCGTCTATTATTTCTGGATGCC | 1 bp transversion of G to C in pBIO1780 to make pBIO1797 |
| TR_G-C_SDMRp1 | GGCATCCAGAAATAATAGACGGTATGGTGTATTAAATCAAGTGCG | 1 bp transversion of G to C in pBIO1780 to make pBIO1797 |
| TR_G-C_SDMFp2 | CGCACTTGATTTAATAGACCATACCGTGTATTATTTCTGGATGCC | 1 bp transversion of C to G in pBIO1780 to make pBIO1798 |
| TR_G-C_SDMRp2 | GGCATCCAGAAATAATACACGGTATGGTCTATTAAATCAAGTGCG | 1 bp transversion of C to G in pBIO1780 to make pBIO1798 |
| acuR_IFDfp1 | GACACCCCTCCATCGGTTCCCCGGCCCGACCCGCTGCAC | Deletion of *acuR* from pBIO1917 to form pBIO1918 |
| acuR_IFDrp1 | GTGCAGCGGGTCGGGCCGGGGAACCGATGGAGGGGTGTC | Deletion of *acuR* from pBIO1917 to form pBIO1918 |
| BambH_EcoRIfp1 | CCGTGGCCG**GAATTC**CGACCGGTG | For cloning fragment used in pBIO1807, pBIO1808 |
| BambH_fusPstI_rp | GCACGC**CTGCAG**CTCGGCGGGGTCCGCC | For cloning fragment used in pBIO1807 |
| BambI_fusPstI_rp | CGCCGAGCTG**CTGCAG**GTAGTCGGCATCG | For cloning fragment used in pBIO1808 |
| Prom_-10for1 | CGCACTTGATTTAATAGACCATACCGTCGCTTACTTCTGGATGCC | Mutation in -10 promoter motif in pBIO1780/ pBIO1917 |
| Prom_-10rev1 | GGCATCCAGAAGTAAGCGACGGTATGGTCTATTAAATCAAGTGCG | Mutation in -10 promoter motif in pBIO1780/ pBIO1917 |
| Prom_-35for1 | CGCACCGAGTTTAATAGACCATACCGTCTATTATTTCTGGATGCC | Mutation in -35 promoter motif in pBIO1780/ pBIO1917 |
| Prom_-35rev1 | GGCATCCAGAAATAATAGACGGTATGGTCTATTAAACTCGGTGCG | Mutation in -35 promoter motif in pBIO1780/ pBIO1917 |
| acuR_5RACE1 | GGCCTTGTTGCGAAAGTAGT | 5′RACE |
| acuR_5RACE2 | GGAACTCTTGCTGCCTTC | 5′RACE |
| RNA oligo A3 | AUAUGCGCGAAUUCCUGUAGAACGAACACUAGAAGAAA | 5′RACE |
| DNA oligo B6 | GCGCGAATTCCTGTAGA | 5′RACE |
| acuR_qPCRf1 | TCTGACAGACACCCCTCCAT | q-RTPCR |
| acuR_qPCRr1 | CTTGCTGCCTTCAGGATCTC | q-RTPCR |
| acuI_qPCRf1 | TCGCCTATTCGACCTTGAAC | q-RTPCR |
| acuI_qPCRr1 | CCCAGCCATTCAGGATGA | q-RTPCR |
| dddL_qPCRf3 | tacggctacgagaaggttcc | q-RTPCR |
| dddL_qPCRr3 | tcgatgtccttgtggctgt | q-RTPCR |
| rpoZ_qPCRf1 | ATCGCGGAAGAGACCCAGAG | q-RTPCR |
| rpoZ_qPCRr1 | GAGCAGCGCCATCTGATCCT | q-RTPCR |
| acuIk19_EcoRIfor1 | GCCGGGCATC**GAATTC**ACGGGCACGG | For cloning fragment used in pBIO1831 |
| acuIk19_PstIrev1 | GGCGGT**CTGCAG**GACCTCGGAGAAGGG | For cloning fragment used in pBIO1831 |
| M13F | CGCCAGGGTTTTCCCAGTCACGAC | Sequencing |
| M13R | TCACACAGGAAACAGCTATGAC | Sequencing |
